# Willingness to be vaccinated against COVID-19: the role of risk perception, trust in institutions, and affects

**DOI:** 10.3389/fpsyg.2023.1182114

**Published:** 2023-09-29

**Authors:** Ghozlane Fleury-Bahi, Arnaud Sapin, Oscar Navarro, Abdel Halim Boudoukha, Jean-Michel Galharret, Amélie Bret, Anne Congard

**Affiliations:** ^1^Laboratoire de Psychologie des Pays de la Loire, Nantes Université, Univ Angers, Nantes, France; ^2^Laboratoire CHROME, Université de Nîmes, Nîmes, France; ^3^Laboratoire de Mathématiques Jean Leray (LMJL), CNRS, Nantes Université, Nantes, France

**Keywords:** COVID-19, risk perception, vaccination, trust in institutions, affects

## Abstract

**Introduction:**

Vaccination is one of the most effective ways to stop the COVID-19 pandemic and prevent severe disease. This study aims to ascertain the determinants of COVID-19 vaccination acceptance in the French population before the vaccine was introduced (France’s second lockdown) and during the roll-out of the vaccination campaign (France’s third lockdown). We focus on the following as determinants of willingness to be vaccinated: risk perception, affects related to the risk, and trust in political and health institutions.

**Method:**

The study was conducted among two convenient samples including 591 and 474 participants. The results show that the timing of the crisis was important. While the intention to be vaccinated was relatively low before the vaccines were introduced, it became significantly higher when the vaccination campaign was deployed.

**Results:**

The results show that risk perception and trust in health institutions are the most relevant predictors of intention to be vaccinated.

**Discussion:**

Results are discussed in terms of the effectiveness of communication campaigns.

## Introduction

Since January 2020, France, like the rest of the world, has been faced with a pandemic linked to the spread of the SARS-COV-2 virus. The COVID-19 pandemic has led France to impose three lockdowns on its population and to ask citizens to adopt barrier measures to limit the spread of the virus. Since 27 December 2020, once the vaccine became available, France has been offering its population the opportunity to be vaccinated, initially giving priority to healthcare workers, people over 70 and those with co-morbidities. It is important in terms of public health policy in the face of a pandemic such as COVID-19 to identify the obstacles and levers to adopting vaccination behavior. This study therefore aims to investigate the role of various determinants of intention to be vaccinated against COVID-19. More specifically, we will address the role played by risk perception, associated emotions and the level of trust in institutions. The focus is on the intention declared during France’s second and third lockdowns. During the second lockdown the vaccine was not yet on the market; during the third, the vaccination campaign was underway, with the vast majority of the population, apart from the youngest, granted the opportunity to be vaccinated.

### Attitudes toward vaccination, vaccine acceptance and vaccine hesitancy

Attitudes toward vaccination can be seen as placed on a continuum ranging from total acceptance to complete refusal (Dubé et al., 2014). Dubé et al. (2014) identified several categories of determinants of vaccine hesitancy: contextual (religious beliefs, rumors…), related to individual or group influences (risk perception, trust in the health system…) and the specificity of the vaccine. A systematic review of influenza vaccine hesitancy (Schmid et al., 2017) reports barriers of vaccination intention at different levels ranging from psychological (e.g., perceived utility of the vaccine; perceived risk of the disease; social benefit) to contextual (e.g., access to vaccines; interaction with the healthcare system) and sociodemographic levels. Betsch et al. (2018) also highlighted that validated measures of vaccine hesitancy principally focus on confidence in vaccines and in the associated system, and secondarily on perceived risk (Betsch et al., 2018). Focusing on COVID-19, another systematic review (Lin et al., 2020) showed that determinants of vaccine acceptance are universal across countries, with subgroups of people with higher levels of education and incomes and the more elderly more likely to get vaccinated (Fisher et al., 2020; Reiter et al., 2020; Mercadante and Law, 2021). The review also identified the importance of perceived severity and infection risk, previous flu vaccination, and trust in government for acceptance of the vaccine against COVID-19.

### Disease risk perception

A key determinant of people’s engagement in vaccination is the level of risk perception related to the disease. Empirical research has shown that the way people perceive risks is a strong predictor of vaccine acceptance. A meta-analysis suggests consistent relationships between risk perceptions and vaccination; more precisely, different dimensions of risk perception like risk likelihood, susceptibility, or perceived severity of the disease significantly predict vaccination behavior or intention (Brewer et al., 2007). People who perceive the risk of contracting the disease as high and consider the consequences of the disease as harmful are more likely to be vaccinated (Brewer et al., 2007; Setbon and Raude, 2010; Thomson et al., 2016; Schmid et al., 2017; Betsch et al., 2018).

Similarly, regarding COVID-19 vaccines, studies have highlighted that higher perceived risk of COVID-19 infection and perceiving COVID-19 as a threat are associated with higher vaccine acceptance (Harapan et al., 2020; Karlsson et al., 2021; Soares et al., 2021). The perceived likelihood of being infected with COVID-19 in the future (Reiter et al., 2020; Khubchandani et al., 2021; Soares et al., 2021) and the perceived severity of a COVID-19 infection are also related to vaccine acceptance (Head et al., 2020; Reiter et al., 2020; Zampetakis and Melas, 2021). Mercadante and Law (2021), using the Health Belief Model, also identified that COVID-19 vaccine intention is directly related to the perceived benefits and perceived barriers regarding the risk. Caserotti et al. (2021) also established links between risk perception and the intention to get vaccinated following the different phases of the COVID-19 emergency in Italy (Caserotti et al., 2021).

### Risk, affects and protective behaviors

Emotional responses to the pandemic have been identified by a number of research studies (Chou and Budenz, 2020; Lwin et al., 2020; Congard et al., 2022), concerning both positive and negative feelings associated with COVID-19. Among the negative outcomes, depressive, anxious and traumatic symptoms were observed in the general population during the lockdown in the USA (Tull et al., 2020), in India (Pandey et al., 2020) or in Italy (Prati, 2020).

Risk perception and affects linked to perceived risk could be associated to vaccination. Indeed, the influence of affects on the adoption of protective behaviors has long been documented in the field of social (Perugini and Bagozzi, 2001) and health psychology (Dillard and Nabi, 2006; Dunlop et al., 2008). The affects are thus examined in the links they have with preventive behaviors (Chapman and Coups, 2006; Peters et al., 2006). Concerning more specifically vaccination behaviors, Chapman and Coups (2006) highlighted that worry and regret were stronger predictors of vaccination than perceived risk and mediated positively the effect of risk on vaccination. In the same vein, unvaccinated individuals experiencing negative emotions about the pandemic were more willing to get the vaccine (Wei et al., 2022). But there is no consensus on the nature of the link between affect and vaccination, since some studies show a negative link between negative affects and intention to receive the vaccine (Berry et al., 2021; Li et al., 2021). These studies focus specifically on affect resulting from perceived uncertainty (Li et al., 2021), misinformation about the COVID-19 vaccine (Berry et al., 2021) or agitation, sadness and anxiety due to the physical distancing measures (Soares et al., 2021). Moreover, Savadori and Lauriola (2021) have highlighted two different roles for affects: negative affective attitude toward COVID-19 positively impacts hygiene and cleaning, whereas affective appraisal of risk mediates these protective behaviors (Savadori and Lauriola, 2021).

Health choices are guided by the anticipation of the affective response to the consequences of the health action (Slovic et al., 2004). For Miller et al. (1996), negative perception of the preventive behavior reduces the likelihood of engaging in the behavior, whereas positive affect is likely to increase it (Miller et al., 1996). The affect heuristic (Slovic and Peters, 2006) explains that information about the benefits of engaging or not engaging in specific health behavior influences the degree of positive or negative affect, which in turn affects risk perception. A vaccination that is said to be of low benefit should provoke negative affects and be evaluated as riskier. Indeed, for Loewenstein et al. (2001), risk perception is not only influenced by cognitions, but also by affective reactions. The literature also suggests that negative affects like anger or fear could promote behavioral change and positively influence health behaviors (Tannenbaum et al., 2015; Carey and Sarma, 2016; MacFarlane et al., 2020).

Beyond affects, risk perception is also influenced by the perception of control over the situation. More specifically, it has been shown that people tend to underestimate risks that are under their control (Weinstein, 1984; Harris, 1996).

### Trust in institutions

Another determinant of vaccine acceptance is the level of trust in institutions. A literature review suggests that trust in health agencies positively influences people’s willingness to adopt protective behavior (Siegrist and Zingg, 2014). Studies have also highlighted that public compliance during a pandemic is diminished by lack of trust in the government. For example, studies show that during the H1N1 pandemic the use of masks was related to the level of trust in the government in Mexico City (Condon and Sinha, 2010), that personal hygiene practices were associated with trust in formal information in Hong Kong (Liao et al., 2011), and that compliance with all the recommended behaviors was associated with trust in the Ministry of Health in Italy (Prati et al., 2011). Regarding the vaccination of infants, Benin (2006) shows that trust, whether in the health institutions or health workers, is also a key determinant of vaccine acceptance among new mothers (Benin, 2006).

When focusing on COVID-19 vaccination, some studies also identify that trust in institutions is a key determinant of vaccine acceptance. In a survey conducted in 19 different countries, participants reporting higher levels of trust in information from government sources were more likely to be vaccinated (Lazarus et al., 2021). Ward et al. (2020) identified, in the French population, that general lack of trust about politics, medicine, science and the pharmaceutical industry is sometimes associated with refusing the COVID-19 vaccine (Ward et al., 2020). Similarly, in Portugal, Soares et al. (2021) identified that low confidence in the health service response during the pandemic, negative perception of government measures, and perception of the information provided as being inconsistent and contradictory were associated with refusal of or delay in vaccination.

The objectives of this article are twofold. The first objective is to study the intention of individuals to be vaccinated or not before the COVID-19 vaccines became available and then during the vaccination campaign itself. The first hypothesis (H1) is that this intention to be vaccinated is not as significant before the arrival of the vaccine compared to when the vaccine has been tested and proven. The second objective is to examine the role of a certain number of variables in the intention to receive the vaccine at both times of this protocol (before the vaccine was introduced, and during the vaccination campaign). The second hypothesis (H2) thus targets the role of risk perception in the intention to receive the vaccination. It is assumed that the greater the perceived risk, the stronger the intention, as identified by studies conducted in various countries around the world (Dror et al., 2020; Reiter et al., 2020; Caserotti et al., 2021; Karlsson et al., 2021; Khubchandani et al., 2021). The third hypothesis (H3) concerns the role of affects: positive affects with a high level of activation (cheerful and delighted) are expected to be positively associated with the intention to be vaccinated, as they enable motivation to action (Peters et al., 2006). The final hypothesis (H4) is that trust in both political and health institutions may be a factor that promotes the intention to receive the vaccine.

The originality of this work is to study simultaneously the three variables of risk perception, trust in institutions and affects in the role that they are likely to play in an individual’s intention to be vaccinated against COVID-19. Moreover, the addition of trust in institutions, is an asset for a better understanding of vaccination behaviors by differentiating between trust in political institutions and trust in health structures. Approaching these variables at two times of measurement, i.e., before the vaccine became available and then during the vaccination campaign, is an asset that improves understanding of the shifts in opinion toward vaccination, and thus allows the study of contextual factors to be combined with psychological parameters of these changes in opinion toward vaccination during the COVID-19 pandemic.

## Materials and methods

### Participants and procedure

The study was conducted among two convenience samples with 591 participants (Sample 1) and 474 participants (Sample 2) after data cleaning. For the first sample, data were collected during France’s second lockdown from November 23 to December 14, 2020, and for the second sample during the third lockdown from April 19 to May 10, 2021. The samples have an average age of Ms1 = 37.0 (SDs1 = 13.4) and Ms2 = 38.3 years (SDs2 = 13.1). Sociodemographic characteristics of the samples are presented in Table 1.

**Table 1:** Sociodemographic characteristics of the samples.

		S1 (*N* = 591)		S2 (*N* = 474)	
		*N*	%	*N*	%
Gender	Female	358	60.6	263	55.5
	Male	160	27.1	156	32.9
	Other/no response	73	12.3	55	11.6
Age	18–30	213	36.0	152	32.1
	31–40	126	21.3	112	23.6
	41–50	85	14.4	85	17.9
	51–60	64	10.8	43	9.1
	Over 60	34	5.8	31	6.5
	No response	69	11.7	51	10.8
Occupation	With professional activity	338	57.2	296	62.4
	Job seekers	34	5.7	31	6.5
	Retired	11	1.9	13	2.7
	Students	66	11.2	33	7.0
	Other	58	9.8	52	11.0
	No response	84	14.2	49	10.4
Education	High school diploma level or less	70	11.8	68	14.3
	Undergraduate	139	23.5	87	18.4
	Graduate	243	41.1	218	46.0
	PhD	55	9.3	52	11.0
	No response	84	14.3	49	10.3

We invited participants to create a code from their personal information in order for us to be able to identify participants who answered twice. These participants were removed so that the samples of this cross-sectional analysis were composed only of different participants.

The data were collected online. Participants were recruited via the social networks of the researchers, using online advertisements or e-mails sent via a variety of media: e-mails and distribution to friends and family, communication from the University of Nantes, solicitation of the French local press, specialized blogs, distribution on social and professional networks (Facebook, Linkedin, Twitter). Participants did not receive any remuneration.

All people under the age of 18 and/or not residing in France were excluded. The questionnaires were completed anonymously via the Qualtrics tool, available on tablet, smartphone and computer. All procedures performed in this study were in accordance with the ethical standards of the Ethics Committee for Non-Interventional Research (CERNI) of Nantes University (ethics committee approval n°19,052,021) and with the 1964 Declaration of Helsinki and its subsequent amendments. Informed consent was obtained from all individual participants included in the study.

### Measures

Data were collected via a questionnaire, including questions on sociodemographic characteristics and socioeconomic status, vaccination, risk perception, affects related to COVID-19, and trust in the institutions.

#### Willingness to be vaccinated against COVID-19

The question of the participants’ willingness to be vaccinated was asked using the following wording: “How willing would you be for you (or someone close to you) to be vaccinated against COVID-19 when vaccines become available?” for the first sample (second lockdown); “How willing would you be for you (or someone close to you) to be vaccinated against COVID-19?” for the second sample (third lockdown); respondents were asked to indicate their opinion on a scale from 0 to 100.

#### Risk perception and perceived control related to the risk of COVID-19

A scale inspired by the psychometric paradigm of Slovic et al. (1985) was used to measure risk perception related to COVID-19. This scale includes six items: three of them relate to perceived vulnerability (e.g., “How concerned are you about the possibility of contracting COVID-19?”), two of them to perceived probability (e.g., “What do you think is the likelihood of a person contracting COVID-19 in your area?) and one item focusing on perceived severity of risk (e.g., “Estimate the severity of a COVID-19 infection, on a scale of 0 to 100”). The scores of perceived vulnerability (3 items), perceived probability of occurrence (2 items) and global risk perception (6 items including perceived vulnerability, probability and severity) were calculated. The reliability is good for the global score of 6 items (Cronbach’s alpha = 0.78 [0.75;80] for T1 and 0.80 [0.78;0.83] for T2) and for the sub-scale of perceived vulnerability (Cronbach’s alpha = 0.81 [0.79;0.84] for T1 and 0.86 [0.84;0.88] for T2). Perceived control related to risk was also measured with one item inspired and by the Brief Illness Perception Questionnaire (Broadbent et al., 2006): “Faced with COVID-19, I have possibilities of personal control, i.e., significant possibilities of action to protect myself.” For the six items of the risk perception scale and for the perceived control item, the participants were asked to answer on a continuous scale from 1 to 100.

#### Affects related to COVID-19

Affects were assessed using the Measurement of Affectivity: Valence/Activation scale (MAVA, Congard et al., 2011). The original scale is made up of 16 items related to 16 affects classified into four subscales according to the level of activation (weak or strong) and the valence (positive or negative) of the affect. Participants have to indicate how they currently feel in the face of the COVID-19 crisis, by responding, for each emotion, on a 0–100% visual analog scale ranging from 1 “Not felt” to 100 “felt intensely.” For this study, the two items which best explain each dimension were selected for a total number of eight items: Activated Negative Affects (ANA) included nervous and worried; Deactivated Negative Affects (DNA) included bored and sad; Activated Positive Affects (APA) included cheerful and delighted; and Deactivated Positive Affects (DPA) included calm and still. Scores for each of the four dimensions were calculated. The four scores are normally distributed whatever the measurement time (no kurtosis or asymmetry coefficients outside −1;1).

### Trust in institutions

The participants’ level of trust in institutions was assessed with a scale created on the basis of the work of various researchers (Poortinga and Pidgeon, 2003). The nine-item tool asks participants to rate on a scale of one to five their degree of trust in the following institutions: the World Health Organization, the European Commission, the French Government, the prefect of the French department of residence, the mayor of the municipality of residence, their Regional Health Agency, scientific experts, heads of medical services and citizens’ associations. An overall score and two sub-scores were calculated: one for health institutions and the other for political institutions. All the scores are normally distributed at both times of measurement. The internal fidelity indicators are satisfactory for both T1 (αtotal = 0.84 [0.82;0.86], αhealth = 0.78 [0.75;0.81], αpolitics = 0.80[0.77;0.83]) and for T2 (αtotal = 0.83 [0.81;0.86], αhealth = 0.75 [0.70;0.78], αpolitics = 0.809 [0.78;0.84]).

### Data analysis

To test the first hypothesis, i.e., to compare the intention to vaccinate before the COVID-19 vaccines were marketed and during the roll-out of the vaccination campaign, *t*-tests were performed to compare levels of risk perception, affects, trust in institutions and willingness to be vaccinated between the two samples. To test the second, third and fourth hypotheses, correlation analyses were conducted to identify links between our variables of interest. To guarantee the validity of these univariate analyses, statistical assumptions were investigated: normality (using skewness and kurtosis indices) and equality of variances (using Levene’s test) (Supplementary Table S1). Data is considered normal if kurtosis and skewness indices are within the interval −1: 1 (Deledalle and Rowe, 2021). Also, a *p*-value correction (Benjamini-Hochberg procedure) was applied for all univariate analyses (Correlation matrix and student’s t) to counter α risk inflation. More precisely, we applied the correction to 3 blocks in distinct ways: the comparison of means tests; the T1 correlations; the T2 correlations. Multiple regression was also conducted with a stepwise procedure on each of the two samples for the outcome of willingness to be vaccinated. In the first step we investigated associations with sociodemographic variables; in the second step regarding risk perception, the different sub-dimensions of risk perception (vulnerability, probability of occurrence and severity), perceived control and perceived exposure were added. In the third step associations with positive and negative affects were investigated. In the final step trust in the institutions was included. Since the models are nested, their differences in performance have been evaluated by calculating Δ*R*^2^, combined with a partial Fisher test to assess the significance of model improvement. Homoscedasticity of the residuals was checked to ensure the validity of these models (Supplementary Figures S1, S2). Finally, three levels of significance are considered: low (0.050 > *p* > 0.010), medium (0.010 > *p* > 0.005) and high (*p* < 0.005).

## Results

### Preliminary analysis

To test the first hypothesis, i.e., to compare the intention to vaccinate before the COVID-19 vaccines were introduced and during the roll-out of the vaccination campaign, mean comparisons allow us to observe the evolution of the variables between the two independent samples, which correspond to two phases of the pandemic, the second and the third lockdowns in France. Concerning the intention to be vaccinated, the results show that the difference is low significant [W(945) = 11.08, *p* = 0.013] with a medium effect size according to Cohen’s standards. Intention to be vaccinated increases significantly between the first period when no vaccine was available and the second that offered the population the possibility to be vaccinated. For all the other variables of interest, there was relative stability in the scores since no Student’s *t* was significant (see Table 2).

**Table 2:** Mean score comparison between T1 and T2.

Variable	T1 (*N* = 591)		T2 (*N* = 474)		Welch’s *t*		
	Mean	SD	Mean	SD	*t*	Cohen’s d	*p*-value
Vaccination^a^	55.6	35.4	79.4	30.8	11.08	0.71	0.013
**Risk perception**							
Vulnerability	35.36	23.90	34.98	24.93	−0.25	−0.02	0.959
Probability of occurrence	56.60	23.00	56.93	22.69	0.23	0.001	0.959
Severity	64.24	28.03	65.64	26.51	0.81	0.05	0.838
Total	46.34	18.73	46.17	19.37	−0.14	−0.01	0.959
Control	58.79	24.39	58.22	24.74	−0.37	−0.02	0.959
**MAVA**							
DPA	56.46	25.472	57.85	24.36	0.88	0.06	0.838
APA	36.70	25.784	37.73	24.54	0.65	0.04	0.838
DNA	52.55	28.710	51.35	27.49	−0.68	−0.04	0.838
ANA	48.40	26.401	45.48	25.62	−1.77	−0.11	0.500
**Trust in institutions**							
Health institutions	3.68	0.903	3.72	0.86	0.83	0.05	0.838
Political institutions	2.39	0.960	2.33	0.93	−0.55	−0.03	0.838
Total	3.04	0.748	3.04	0.73	0.01	>0.01	0.996

a Welch *t*-test was conducted instead of student *t*-test due to a heterogeneity of variances (see Supplementary Table S1).

MAVA, measurement of affectivity: valence/activation; DNA, deactivated negative affect; ANA, activated negative affect; DPA, deactivated positive affect; APA: activated positive affect.

To test H2, H3, and H4, correlations showed that the willingness to be vaccinated is positively and significantly correlated with trust in institutions for both periods, with the different measures of risk and perceived control in the second period only, and with Activated Negative Affects (ANA) for both periods (see Table 3).

**Table 3:** Matrix of Pearson’s correlations for T1 and T2.

	Vaccination T1		Vaccination T2	
	*r* [CI95%]	*p*-value	*r* [CI95%]	*p*-value
**Risk perception**				
Vulnerability	0.107 [0.021;0.191]	0.033	0.148 [0.054;0.240]	0.004
Probability of occurrence	−0.013 [−0.098;0.073]	0.842	0.119 [0.024;0.212]	0.021
Severity	0.128 [0.043;0.212]	0.009	0.132 [0.037;0.224]	0.012
Control	0.029 [−0.016;0.154]	0.668	0.170 [0.076;0.261]	0.002
Total	0.070 [−0.056;0.115]	0.192	0.178 [0.084;0.269]	0.002
**MAVA**				
DPA	−0.005 [−0.091;0.081]	0.906	−0.069 [−0.163;0.027]	0.191
APA	−0.045 [−0.130;0.041]	0.451	0.000 [−0.095;0.096]	0.994
DNA	0.019 [−0.066;0.105]	0.789	0.031 [−0.065;0.126]	0.576
ANA	0.086 [0.000;0.170]	0.100	0.116 [0.020;0.209]	0.022
**Trust in institutions**				
Health institutions	0.443 [0.371;0.509]	0.004	0.430 [0.349;0.504]	0.002
Political institutions	0.263 [0.182;0.341]	0.004	0.292 [0.202;0.377]	0.002
Total	0.373 [0.297;0.444]	0.004	0.380 [0.295;0.458]	0.002

MAVA, measurement of affectivity: valence/activation; DNA, deactivated negative affect; ANA, activated negative affect; DPA, deactivated positive affect; APA, activated positive affect.

### Main determinants of intention to be vaccinated against COVID-19

For the outcome of willingness to be vaccinated, hierarchical multiple regression was conducted with a stepwise procedure. The first model (Model 0) included the variables of gender, age, education and occupation. In the second model (Model 1), variables related to perceived risk (perceived vulnerability, perceived severity, perceived probability of occurrence, perceived control and exposure to the risk) were added to the previous ones. In the third model (Model 2), the four affect scores were included. Finally, in Model 3, the two scores of trust in institutions (health institutions and political institutions) were also considered. Tables 4, 5 present the results of the hierarchical multiple regression. The parameters confidence intervals of all models are available in Supplementary Table S2.

**Table 4:** Hierarchical regression analyses for blocks of variables predicting vaccination intentions in T1.

	M0		M1		M2		M3	
	β	*p*-value	**β**	*p*-value	**β**	*p*-value	**β**	*p*-value
(Intercept)	0.000	<0.001	0.000	<0.001	0.000	0.137	0.000	<0.001
Gender = Female	0.475	<0.001	0.513	<0.001	0.516	<0.001	0.516	<0.001
**Occupation = Employed**								
Unemployed	0.145	0.438	0.093	0.617	0.139	0.456	0.177	0.291
Student	0.212	0.131	0.186	0.179	0.188	0.177	0.137	0.273
Retired	−0.009	0.956	−0.023	0.881	0.031	0.839	−0.085	0.544
Other	0.155	0.553	0.071	0.784	0.052	0.840	−0.082	0.725
Age	−0.075	0.105	−0.088	0.060	−0.082	0.081	0.070	0.130
**Education = High school diploma level**								
High school diploma+2 yrs	0.183	0.225	0.177	0.238	0.189	0.206	0.188	0.161
High school diploma+5 yrs	0.029	0.837	0.017	0.906	0.039	0.782	0.069	0.583
High school diploma+8 yrs	0.075	0.698	0.102	0.594	0.131	0.493	0.223	0.195
Alone = YES	0.002	0.985	0.010	0.936	<0.001	0.995	0.016	0.879
Vulnerability			0.147	0.003	0.106	0.048	0.090	0.064
Probability of occurrence			−0.042	0.442	−0.043	0.427	−0.047	0.340
Severity			0.127	0.005	0.131	0.004	0.112	0.006
Control			0.027	0.619	0.033	0.542	−0.013	0.784
DPA					0.130	0.073	0.048	0.460
APA					−0.063	0.292	−0.047	0.393
DNA					−0.063	0.324	−0.036	0.528
ANA					0.202	0.007	0.193	0.005
Health trust							0.433	<0.001
Political trust							0.037	0.432
*F*	3.54		3.87		3.46		9.20	
Model’s *p*-value	<0.001		<0.001		<0.001		<0.001	
*R* ^2^	0.072		0.108		0.123		0.293	

MAVA, measurement of affectivity: valence/activation; DNA, deactivated negative affect; ANA, activated negative affect; DPA, deactivated positive affect; APA, activated positive affect.

**Table 5:** Hierarchical regression analyses for blocks of variables predicting vaccination intentions in T2.

	M0		M1		M2		M3	
	β	*p*-value	**β**	*p*-value	**β**	*p*-value	**β**	*p*-value
(Intercept)	0.000	<0.001	0.000	<0.001	0.000	0.001	0.000	0.122
Gender = Female	0.269	0.011	0.286	0.006	0.293	0.006	0.251	0.008
**Occupation = Employed**								
Unemployed	−0.149	0.455	−0.085	0.668	−0.094	0.637	−0.104	0.557
Student	0.069	0.725	0.070	0.714	0.056	0.773	−0.048	0.780
Retired	−0.082	0.634	−0.096	0.570	−0.099	0.559	−0.135	0.369
Other	−0.091	0.722	−0.098	0.697	−0.101	0.689	−0.293	0.192
Age	0.159	0.002	0.085	0.122	0.105	0.066	0.220	<0.001
**Education = High school diploma level**								
High school diploma+2 yrs	−0.074	0.661	−0.094	0.575	−0.118	0.485	−0.173	0.247
High school diploma+5 yrs	−0.088	0.566	−0.075	0.617	−0.088	0.563	−0.146	0.282
High school diploma+8 yrs	−0.055	0.786	−0.098	0.626	−0.120	0.554	−0.237	0.187
Alone = YES	−0.114	0.424	−0.107	0.442	−0.088	0.531	0.012	0.924
Vulnerability			0.090	0.127	0.052	0.425	0.027	0.640
Probability of occurrence			0.025	0.674	0.024	0.689	−0.011	0.832
Severity			0.145	0.005	0.145	0.006	0.104	0.026
Control			0.127	0.043	0.122	0.054	0.081	0.148
DPA					−0.005	0.994	−0.036	0.606
APA					0.053	0.410	0.037	0.517
DNA					0.030	0.673	0.059	0.349
ANA					0.072	0.367	0.062	0.384
Health trust							0.452	<0.001
Political trust							0.040	0.441
*F*	1.72		2.75		2.25		7.65	
Model’s *p*-value	0.075		<0.001		0.003		<0.001	
*R* ^2^	0.044		0.095		0.101		0.299	

MAVA, measurement of affectivity: valence/activation; DNA, deactivated negative affect; ANA, activated negative affect; DPA, deactivated positive affect; APA, activated positive affect.

The regression analyses revealed that, with the first model M0, the variable of gender (T1: *R*^2^ = 0.72, *b* = 0.475, *p* = <0.001; T2: *R*^2^ = 0.44, *b* = 0.269, *p* = <0.001) contributed highly and significantly to the prediction of willingness to be vaccinated, associated to the variable of age (*b* = 0.159, *p* = <0.001) for T2. Men are more likely to be motivated to receive the vaccine both before and after its introduction, and older people once it was available.

Introducing perceived risk variables (Model 1) significantly and highly changes the *R*^2^ for T1 (*R*^2^ = 0.108, Δ*R*^2^ = 0.035, *p* = 0.001) and T2 (*R*^2^ = 0.095, Δ*R*^2^ = 0.051, *p* = 0.001). In T1, before the vaccine was available, perceived vulnerability (*b* = 0.147; *p* = <0.001) and perceived severity (*b* = 0.111, *p* = 0.007), contribute significantly and moderately to explaining willingness to be vaccinated. In T2, perceived severity (*b* = 0.143, *p* = 0.005) and perceived control (*b* = 0.127, *p* = 0.04) contribute significantly and highly to explaining vaccination. The higher the levels of perceived severity, control and vulnerability, the higher the willingness to be vaccinated.

When the four scores of emotion were added to the model (Model 2), the *R*^2^ does not change significantly in T1 (*R*^2^ = 0.123, Δ*R*^2^ = 0.015, *p* = 0.106) and T2 (*R*^2^ = 0.101, Δ*R*^2^ = 0.005, *p* = 0.52). So, introducing emotion indicators does not make the model more significant. Nevertheless, in T1, activated negative affects (ANA) significantly and moderately explain willingness to be vaccinated (*b* = 0.202, *p* = 0.07). Before the introduction of the vaccine, the more the participants feel nervous and worried, the more they feel motivated to be vaccinated. We no longer find this effect of negative affects once the vaccine is available (*b* = 0.072, *p* = 0.367). Finally, the addition of the two scores of trust in the institutions (Model 3) made it possible to highly and significantly improve the fit of the model in T1 (*R*^2^ = 0.293, Δ*R*^2^_m_ = 0.171, *p* = <0.001) and T2 (*R*^2^ = 0.299, Δ*R*^2^_m_ = 0.198, *p* = <0.001), with a highly significant regression coefficient for trust in health institutions in T1 (*b* = 0.433, *p* = <0.001) and T2 (*b* = 0.452, p = <0.001). This result shows that the level of trust in health institutions contributes significantly to vaccine acceptance.

In T1, the complete model shows the role of gender (*b* = 0.516; *p* = <0.001), perceived severity of risk (*b* = 0.112, *p* = 0.006), activated negative affects (*b* = 0.193, *p* = <0.005), and trust in health institutions (*b* = 0.433, *p* = <0.001) in willingness to be vaccinated. In T2, the complete model highlights that gender (*b* = 0.251, *p* = 0.008), age (*b* = 0.220, *p* = <0.001), perceived severity of risk (*b* = 0.104, *p* = 0.026) and trust in health institutions (*b* = 0.452, *p* = <0.001) are significantly related to vaccine acceptance.

As regards T1, we firstly observe that all the models significantly predict the variance of the motivation to be vaccinated. However, while the proportion of variance explained is relatively low for the first three models (*R*^2^ of 0.072–0.123), there is a 2.1% increase in explained variance with the addition of trust in institutions. As in T1, in T2 the first three models are weakly effective (*R*^2^ = 0.044–0.101), while the full model explains 29.9% of the variance of intention to be vaccinated.

## Discussion

The aim of this paper was to investigate how risk perception, affects related to risk and trust in institutions could explain COVID-19 vaccination acceptance in the French population before the vaccine was available and during the deployment of the vaccination campaign. For the two periods we hypothesized that these variables contribute to explaining the intention to be vaccinated against COVID-19 and that the level of vaccine acceptance is positively linked with perceived risk, trust in institutions and positive affects. We also hypothesized higher levels of willingness to be vaccinated in the second period, after COVID-19 vaccines were introduced.

Regarding this first hypothesis, results show that participants reported average levels of intention to be vaccinated before the vaccine was rolled out, and this level significantly and highly increases when vaccination becomes possible, to reach a high level of intention in the second period. At Time 1, the clinical tests have not been finalized and the vaccine promotion campaign has not begun, whereas at Time 2, the efficacy of the vaccine has been proven scientifically and the vaccine promotion campaign is being deployed in the media, among other factors that may encourage the intention to be vaccinated.

Regarding our second objective, the complete models for T1 and T2 highlight the association between vaccination acceptance and gender, perceived severity of COVID-19, and trust in health institutions. More precisely, the effect of these three variables is identified before and after vaccination was deployed. The level of vaccination acceptance is significantly higher for men, and when the levels of perceived severity of the risk and trust in health institutions increase. The overall models do not allow us to identify a significant role of any of the negative or positive affects. Considering the two periods, we observe that gender, perception of severity of risk, and trust in health institutions are stable predictors over time of the willingness to be vaccinated.

With the first model (Model 0), we investigated the links between vaccine acceptance and sociodemographic variables, and highlighted that the willingness to be vaccinated is greater for men whatever the period, and for older people after the vaccine is rolled out. Differences in willingness to receive the vaccine against COVID-19 across gender have already been identified in the literature. Neumann-Böhme et al. (2020) found significant differences in willingness to be vaccinated across gender, with a significantly higher proportion of men who intend to get vaccinated (Neumann-Böhme et al., 2020), while Khubchandani et al. (2021) pinpointed more vaccine hesitancy among women (Khubchandani et al., 2021). Our results could be explained by the greater vulnerability of men to the virus (Bwire, 2020). Older people are also more vulnerable to COVID-19 with increased risk for serious complications from viral infection and are thus more willing to be vaccinated than younger people, for whom the health consequences are slighter (Soares et al., 2021).

Regarding the role of risk perception (H2; Model 1), we investigated the link between risk perception and the intention to be vaccinated, and identified that perceived severity of risk and perceived vulnerability are the most significant variables to explain vaccine acceptance before the vaccine was introduced, and perceived severity and perceived control, after its deployment. As hypothesized, these results show that the way the risk is perceived in terms of severity of the consequences and personal vulnerability is a major determinant of intention to be vaccinated against COVID-19 whatever the period. The intention to be vaccinated increases with risk perception. Previous research has already shown that the more serious the risk is perceived to be, and the more individuals consider themselves at risk of disease, the more likely they are to accept the vaccine. This has been shown for COVID-19 in the general population in Italy (Caserotti et al., 2021), Finland (Karlsson et al., 2021), the United States (Reiter et al., 2020; Khubchandani et al., 2021) and for healthcare workers (Dror et al., 2020) and also for other vaccines such as flu (Freimuth et al., 2017).

Regarding the role of affect (H3), we investigated with Model 2 the link between affects and intentions to be vaccinated against COVID-19. Despite the fact that introducing the four scores of emotion did not make the model significant, it was shown that the score of activated negative affects (including both nervous and worried affects) contributes significantly to the explanation of willingness to be vaccinated before the vaccination was deployed, but not afterwards (Chou and Budenz, 2020). These results do not support our hypothesis. Nor do they support the findings of certain studies that suggest that positive affects may be a motivating factor in vaccination intentions (Chapman and Coups, 2006; Peters et al., 2006). The fact that negative affects no longer play a role during the implementation of the vaccination campaign can be explained by the fact that at the time of the vaccination promotion campaign, the French wanted to leave the crisis and the repeated lockdowns (three in France) behind them. Their intention to be vaccinated was perhaps more related to this motivation than to any positive or negative affects.

To test H4, with Model 3 we explored the links between trust in health and political institutions and willingness to be vaccinated. The results show that the level of trust in health institutions contributes significantly to vaccine acceptance both before and after the roll-out of the vaccine. Trust in health institutions is the factor that best explains willingness to be vaccinated. We had also hypothesized a link with political institutions, but our results do not identify this in the way some studies do (Lazarus et al., 2021; Soares et al., 2021). The role played by trust in health institutions in take-up of vaccination has already been identified in some studies. For instance, Soares et al. (2021), in a community-based survey in Portugal, show that low confidence in the health service during the pandemic is associated with refusal of or delay in vaccination. Likewise, Liu and Chu (2022) found among US participants that trust in healthcare providers, public health agencies and pharmaceutical companies led to a more positive attitude toward the COVID-19 vaccine (Liu and Chu, 2022). In contrast, trust in politicians did not play a role in vaccination intention. These results are in line with our findings that trust in health institutions and not in political institutions explains the intention to vaccinate.

Trust is a major factor in the acceptance of protective measures related to risks perceived as uncontrollable in terms of personal exposure (Frewer, 2004). Information coming from a source considered as reliable is internalized and determines protective reactions. Inversely, information coming from sources considered suspicious, unreliable or promoting particular interests can generate opposition and consequences opposite to those expected. Our result can be explained by the different sources of trust involved here, with on the one hand, trust based on the expertise of institutions, i.e., trust based on competence, and on the other hand trust based on the perceived honesty of the institution, i.e., trust based on integrity (Terwel et al., 2009). Regarding our results, it would therefore seem that it is trust in competence regarding health institutions that is more important than the perceived integrity of political institutions.

This research is not without limitations. First, we did not reach representative samples: we used snowball sampling techniques, which can skew the features of the sample. Secondly, participants with higher levels of education are over-represented compared to the general population. It’s also important to note that the links between risk perception and intention to vaccinate are significant but fairly weak. So the results must be interpreted carefully. Furthermore, to compare willingness to be vaccinated before and after vaccines were deployed, we used two different samples. Working with the same participants would have allowed us to control the intention to be vaccinated in T1 and see the impact of other variables in T2. Moreover, introduction of other variables, such as confidence in vaccines or beliefs about vaccination, would have made it possible to achieve a higher level of explained variance. We also found that the vaccine variable did not quite follow a normal distribution (Supplementary Table S1), which probably had an effect on the analyses, especially the regression models. Many other questions have yet to be answered, and can be explored statistically using these data and notably the mediating role of affect between trust in institutions and the perception of risk regarding the intention to be vaccinated.

## Conclusion

Our results have implications for risk and crisis communication during a pandemic situation. They could, in consequence, contribute to vaccination promotion campaign in different ways. First, to communicate effectively about the pandemic risk and protective behaviors, it is important to involve the various health stakeholders in order to increase the level of trust in them. Siegrist and Zingg (2014) emphasize that a transparent information strategy should be used in order to foster trust in health authorities. Risk communication is also important to promote better knowledge of the risk, since we have shown that the perceived seriousness of the risk promotes acceptance of the vaccine. Our results could also contribute to research because they emphasize that research gaps should be addressed to better understand the role of trust when dealing with pandemics (Siegrist and Zingg, 2014). Consequently, future research directions could for example focus on the determinants of trust in institutions to better understand the links between trust, risk perception and protective behaviors, as this variable seems particularly important in a pandemic context.

## Data availability statement

The data that support the findings of this study are available at: https://osf.io/a7nkz/.

## Author contributions

GF-B, AC, ABo, ABr, and ON designed the survey and contributed to its implementation. GF-B wrote the manuscript with support from AS and AC. AS and J-MG carried out the statistical analyses. All authors contributed to the article and approved the submitted version.
