# Supplementary material for: Willingness to be vaccinated against COVID-19: the role of risk perception, trust in institutions, and affects
Source: Front Psychol. 2023 Sep 29;14:1182114. doi: 10.3389/fpsyg.2023.1182114 (PMC10576432; doi:10.3389/fpsyg.2023.1182114)
Supplement: Supplementary file 1 [file Data_Sheet_1.docx]

SUPPLEMENTARY MATERIAL

Table 1 : Evaluation of the variables of interest normality and the equality of variances between times (Levene's test)

| Variable | T1 (N=591) | | T2 (N=474) | | Levene’s test | |
| --- | --- | --- | --- | --- | --- | --- |
|  | Skewness | Kurtosis | Skewness | Kurtosis | F | p-value |
| Vaccination | -.280 | -1.279 | -1.4500 | .846 | 26.512 | < .001 |
| Risk perception |  |  |  |  |  |  |
| Vulnerability | .536 | -.502 | .494 | -.692 | 1.976 | .160 |
| Probability of occurrence | -.160 | -.652 | -.233 | -.572 | 0.356 | .551 |
| Severity | -.709 | -.385 | -.730 | -.238 | 2.250 | .134 |
| Total | .231 | -.270 | .258 | -.457 | .810 | .368 |
| Control | -.358 | -.577 | -.290 | -.625 | .017 | .895 |
| MAVA |  |  |  |  |  |  |
| DPA | -.153 | -.782 | -.269 | -.687 | 1.532 | .216 |
| APA | .444 | -.479 | .354 | -.436 | 1.284 | .257 |
| DNA | -.328 | -.975 | -.204 | -.934 | 1.326 | .250 |
| ANA | -.102 | -.838 | .043 | -.967 | .150 | .698 |
| Trust in institutions |  |  |  |  |  |  |
| Health institutions | -.828 | .413 | -.719 | .249 | 1.211 | .271 |
| Political institutions | .436 | -.471 | .580 | -.154 | .254 | .614 |
| Total | -.341 | .082 | .077 | -.020 | .298 | .585 |

SUPPLEMENTARY MATERIAL

Figure
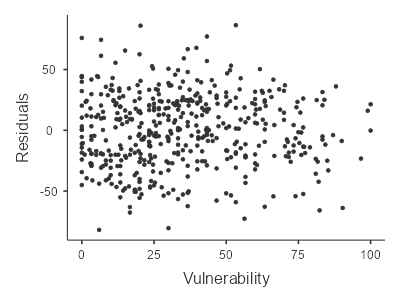

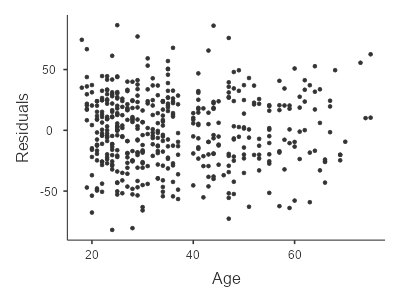
1 : Graphical representation of residual homoscedasticity (Model 4 - T1)


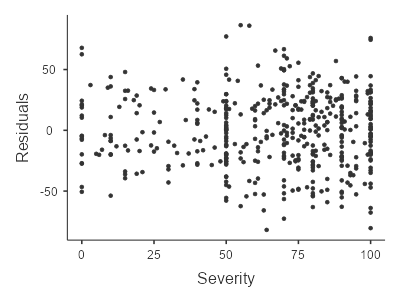

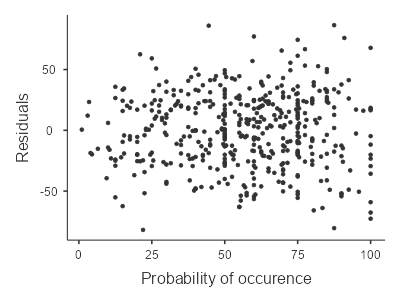

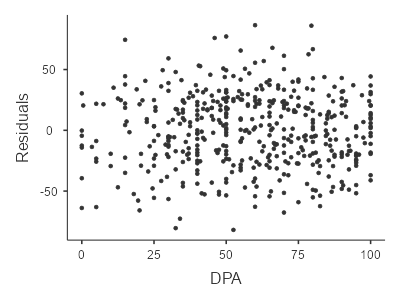

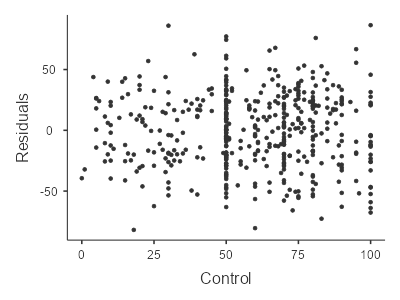


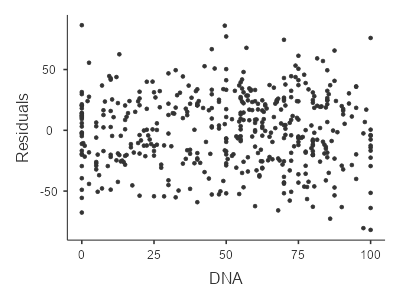

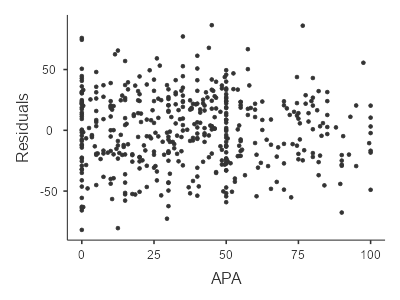


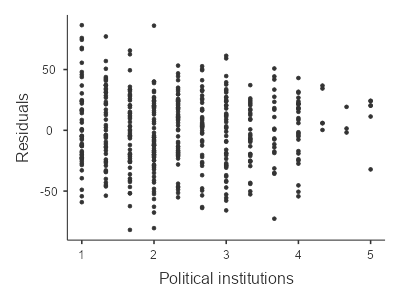

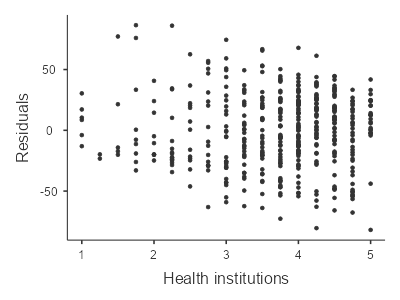

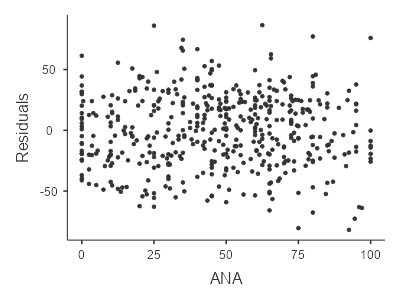


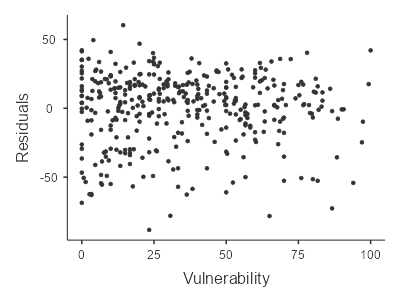

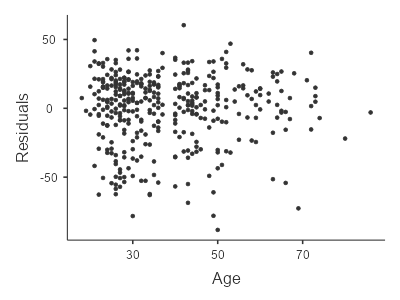
SUPPLEMENTARY MATERIAL

Figure 2 : Graphical representation of residual homoscedasticity (Model 4 – T2)


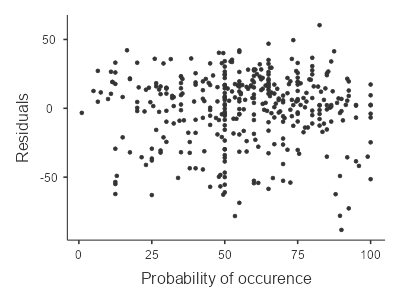


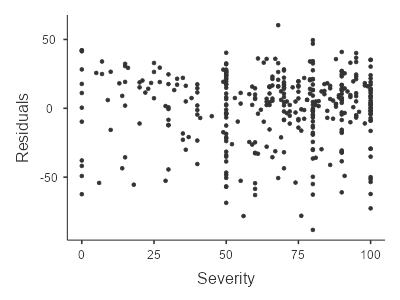


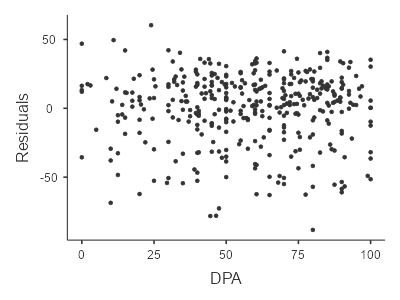

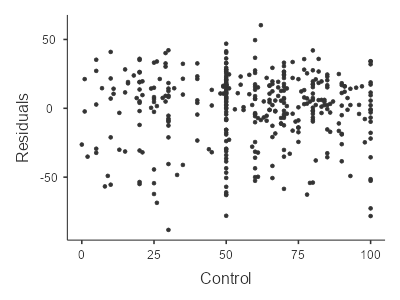


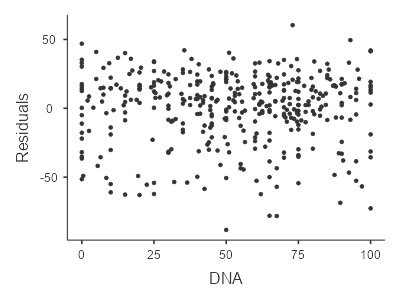

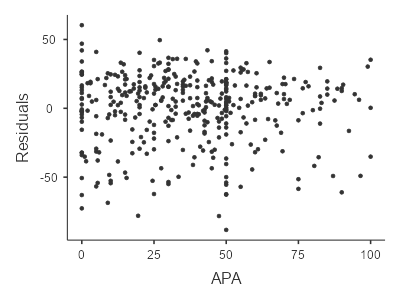


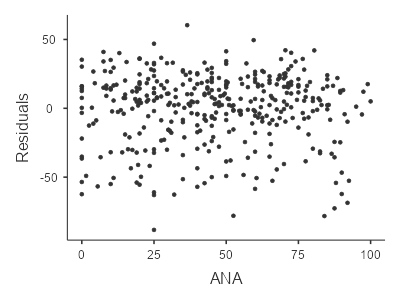


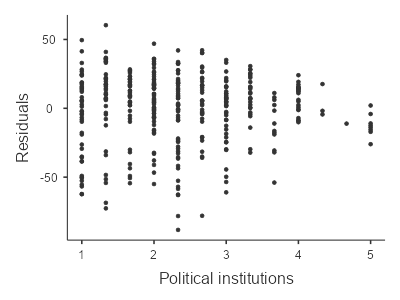


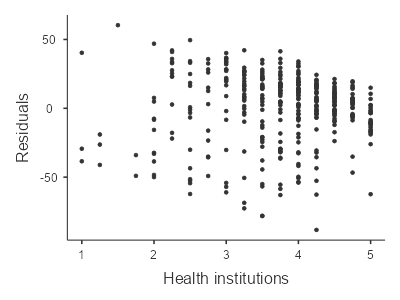


SUPPLEMENTARY MATERIAL

Table 2 : Confidence interval of estimated parameters in multiple linear regression models

| **Predictors** | **T1: IC95% of β** | | | | **T2: IC95% of β** | | | |
| --- | --- | --- | --- | --- | --- | --- | --- | --- |
|  | M0 | M1 | M2 | M3 | M0 | M1 | M2 | M3 |
| (Intercept) |  |  |  |  |  |  |  |  |
| Gender=Female | [.28;.67] | [.32;.71] | [.32;.71] | [.34;.69] | [.06;.48] | [.08;.49] | [.08;.5] | [.07;.44] |
| Occupation = Employed |  |  |  |  |  |  |  |  |
| Unemployed | [-.22;.51] | [-.27;.46] | [-.23;.5] | [-.15;.51] | [-.54;.24] | [-.47;.3] | [-.49;.3] | [-.45;.24] |
| Student | [-.06;.49] | [-.09;.46] | [-.08;.46] | [-.11;.38] | [-.31;.45] | [-.31;.45] | [-.32;.44] | [-.38;.29] |
| Retired | [-.31;.29] | [-.32;.28] | [-.27;.34] | [-.36;.19] | [-.42;.26] | [-.43;.24] | [-.43;.24] | [-.43;.16] |
| Other | [-.36;.67] | [-.44;.58] | [-.46;.56] | [-.54;.38] | [-.59;.41] | [-.59;.4] | [-.6;.4] | [-.73;.15] |
| Age | [-.17;.02] | [-.18;0] | [-.17;.01] | [-.02;.16] | [.06;.26] | [-.02;.19] | [-.01;.22] | [.12;.32] |
| Education = High school diploma level |  |  |  |  |  |  |  |  |
| High school diploma+2 yrs | [-.11;.48] | [-.12;.47] | [-.1;.48] | [-.08;.45] | [-.41;.26] | [-.42;.23] | [-.45;.21] | [-.47;.12] |
| High school diploma+5 yrs | [-.25;.31] | [-.26;.29] | [-.24;.31] | [-.18;.32] | [-.39;.21] | [-.37;.22] | [-.39;.21] | [-.41;.12] |
| High school diploma+8 yrs | [-.3;.45] | [-.27;.48] | [-.24;.51] | [-.11;.56] | [-.46;.35] | [-.49;.3] | [-.52;.28] | [-.59;.12] |
| Alone=YES | [-.23;.24] | [-.22;.24] | [-.23;.23] | [-.19;.23] | [-.39;.17] | [-.38;.17] | [-.36;.19] | [-.23;.26] |
| Vulnerability |  | [.05;.24] | [0;.21] | [-.01;.18] |  | [-.03;.2] | [-.08;.18] | [-.09;.14] |
| Probability of occurrence |  | [-.15;.06] | [-.15;.06] | [-.14;.05] |  | [-.09;.14] | [-.09;.14] | [-.12;.09] |
| Severity |  | [.04;.22] | [.04;.22] | [.03;.19] |  | [.04;.25] | [.04;.25] | [.01;.2] |
| Control |  | [-.08;.13] | [-.07;.14] | [-.11;.08] |  | [0;.25] | [0;.25] | [-.03;.19] |
| DPA |  |  | [-.01;.27] | [-.08;.18] |  |  | [-.16;.15] | [-.17;.1] |
| APA |  |  | [-.18;.05] | [-.15;.06] |  |  | [-.07;.18] | [-.08;.15] |
| DNA |  |  | [-.19;.06] | [-.15;.08] |  |  | [-.11;.17] | [-.06;.18] |
| ANA |  |  | [.05;.35] | [.06;.33] |  |  | [-.08;.23] | [-.08;.2] |
| Health trust |  |  |  | [-.05;.13] |  |  |  | [-.06;.14] |
| Political trust |  |  |  | [.34;.53] |  |  |  | [.35;.55] |
| **F** | 1.72 | 2.75 | 2.25 | 7.65 | 3.54 | 3.87 | 3.46 | 9.20 |
| **Model’s p-value** | .075 | <.001 | .003 | <.001 | <.001 | <.001 | <.001 | <.001 |
| **R²** | .044 | .095 | .101 | .299 | .072 | .108 | .123 | .293 |
